# Ethyl 4-(4-hydroxy­phen­yl)-6-methyl-2-oxo-1,2,3,4-tetra­hydro­pyrimidine-5-carboxyl­ate monohydrate

**DOI:** 10.1107/S1600536809037441

**Published:** 2009-09-19

**Authors:** Susanta K. Nayak, K. N. Venugopala, Deepak Chopra, Thavendran Govender, Hendrik G. Kruger, Glenn E. M. Maguire, T. N. Guru Row

**Affiliations:** a Solid State and Structural Chemistry Unit, Indian Institute of Science, Bangalore 560 012, India; b School of Chemistry, University of KwaZulu-Natal, Durban 4000, South Africa; c Department of Chemistry, Indian Institute of Science Education and Research, Bhopal 462 023, India; d School of Pharmacy and Pharmacology, University of Kwazulu-Natal, Durban 4000, South Africa

## Abstract

In the title compound, C_14_H_16_N_2_O_4_·H_2_O, the dihedral angles between the planes of the 4-hydroxy­phenyl and ester groups with the plane of the six-membered tetra­hydro­pyrimidine ring are 87.3 (1) and 75.9 (1)°, respectively. The crystal structure is stabilized by O—H⋯O and N—H⋯O hydrogen bonding between the water mol­ecule and the organic functionalities.

## Related literature

Bignelli compounds are poly-functionalized dihydro­pyrimidines exhibiting a broad range of therapeutic and pharmacological properties, see: Atwal *et al.* (1991 ▶); Jauk *et al.* (2000 ▶); Kappe (2000 ▶); Kato (1984 ▶).
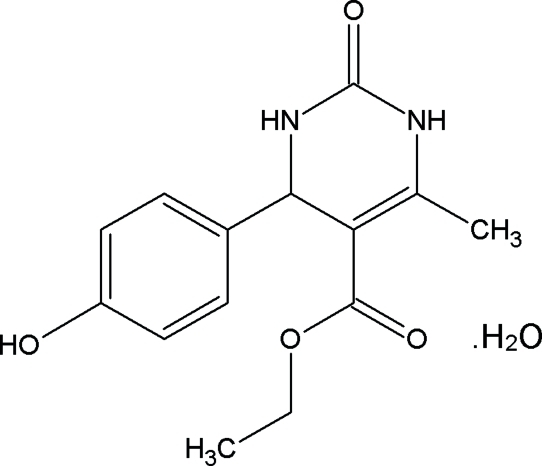


## Experimental

### 

#### Crystal data

C_14_H_16_N_2_O_4_·H_2_O
*M* *_r_* = 294.30
Triclinic, 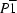

*a* = 5.6859 (2) Å
*b* = 10.7190 (5) Å
*c* = 12.1980 (5) Å
α = 85.267 (3)°
β = 83.990 (3)°
γ = 74.936 (4)°
*V* = 712.76 (6) Å^3^
*Z* = 2
Mo *K*α radiation
μ = 0.11 mm^−1^
*T* = 292 K
0.38 × 0.24 × 0.15 mm

#### Data collection

Goniometer Xcalibur with Eos (Nova) detector diffractometer
Absorption correction: multi-scan (*CrysAlis Pro*; Oxford Diffraction, 2009 ▶) *T*_min_ = 0.951, *T*_max_ = 0.984
18207 measured reflections
2792 independent reflections
2109 reflections with *I* > 2σ(*I*)
*R*_int_ = 0.034

#### Refinement

*R*[*F* ^2^ > 2σ(*F* ^2^)] = 0.037
*wR*(*F* ^2^) = 0.102
*S* = 1.09
2792 reflections
201 parameters
H atoms treated by a mixture of independent and constrained refinement
Δρ_max_ = 0.19 e Å^−3^
Δρ_min_ = −0.14 e Å^−3^

### 

Data collection: *CrysAlis Pro* (Oxford Diffraction, 2009 ▶); cell refinement: *CrysAlis Pro*; data reduction: *CrysAlis Pro*; program(s) used to solve structure: *SHELXL97* (Sheldrick, 2008 ▶); program(s) used to refine structure: *SHELXL97* (Sheldrick, 2008 ▶); molecular graphics: *ORTEP-3 for Windows* (Farrugia, 1997 ▶) and *CAMERON* (Watkin *et al.*, 1993 ▶); software used to prepare material for publication: *PLATON* (Spek, 2009 ▶).

## Supplementary Material

Crystal structure: contains datablocks global, I. DOI: 10.1107/S1600536809037441/hg2566sup1.cif

Structure factors: contains datablocks I. DOI: 10.1107/S1600536809037441/hg2566Isup2.hkl

Additional supplementary materials:  crystallographic information; 3D view; checkCIF report

## Figures and Tables

**Table 1:** Hydrogen-bond geometry (Å, °)

*D*—H⋯*A*	*D*—H	H⋯*A*	*D*⋯*A*	*D*—H⋯*A*
N1—H1⋯O1^i^	0.86	2.09	2.9411 (17)	171
N2—H2⋯O4^ii^	0.86	2.14	2.978 (2)	165
O4—H4⋯O5*W*^iv^	0.82	1.86	2.674 (2)	176
O5*W*—H1*W*⋯O2	0.83 (3)	2.06 (3)	2.881 (2)	172 (3)
O5*W*—H2*W*⋯O1^iii^	0.93 (3)	1.88 (3)	2.799 (2)	167 (2)

Symmetry codes: (i) 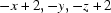; (ii) 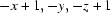; (iii) 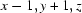; (iv) 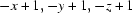.

**Fig. 1.**
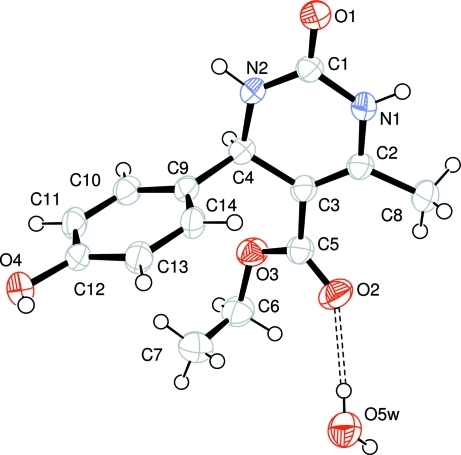
: Molecular structure shows the atom labelling scheme with displacement ellipsoids for non-H atoms at 50% probability level. The dotted line shows the O—H···O intramolecular interaction.

**Fig. 2.**
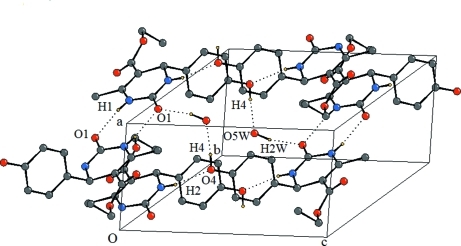
: The molecular packing depicting intermolecular N—H···O and O—H···O hydrogen bonds.

## supplementary crystallographic information

### Comment

Bignelli compounds are poly-functionalized dihydropyrimidine (DHPM) exhibiting a broad range of therapeutic and pharmacological properties (Kappe, 2000), namely, anticarcinogenic (Kato, 1984), antihypertensive (Atwal *et al.*, 1991) and calcium channel modulators (Jauk *et al.*, 2000, and references therein). It is observed that the six-membered tetrahydropyrimidine ring exists in a nearly planar conformation (Figure 1). The ester moiety is in an s-*trans* conformation with respect to the endocyclic double bond. The water molecule is held by O—H···O hydrogen bond, involving H1W with the oxygen of the ester carbonyl moiety. The other hydrogen atom H2W forms intermolecular O—H···O hydrogen bond with the carbonyl group of the tetrahydropyrimidine ring, thereby acting as a bridge between two molecules. Furthermore, the amino hydrogen H1 forms centrosymmetric N—H···O dimers. The other acidic hydrogen H2 forms N—H···O hydrogen bond with the phenolic oxygen atom. The phenolic hydrogen in turn forms O—H···O intermolecular hydrogen bond with the oxygen of the water molecule (Figure 2).

### Experimental

A mixture of ethylacetoacetate (0.1 mol), *para* hydroxy substituted benzaldehyde (0.1 mol) and urea was refluxed in 50.0 mL of ethanol for 2.0 hrs in presence of concentrated hydrochloric acid as catalyst. The reaction completion was monitored through thin layer chromatography and the reaction mixture was quenched in ice cold water. The precipitate obtained was filtered, dried and crystallized from methanol to obtain the title compound.

### Refinement

The hydrogen atoms of the water molecule were located from a difference Fourier map and refined isotropically. The O—H bond lengths are in the range of 0.83 (3)—0.93 (3) Å. The remaining H atoms were positioned geometrically, with C—H = 0.93 Å, 0.96 Å, 0.97 Å, 0.98Å for aromatic, methyl, methylene and methine H atoms respectively and N—H = 0.86Å for amino H atoms and all refined using a riding model with *U*_iso_(H)= 1.2 *U*_eq_(C, N) for aromatic and amine hydrogen and 1.5 *U*_eq_(C) for methyl, methylene and methine H atoms respectively.

### Crystal data

**Table:**

C_14_H_16_N_2_O_4_·H_2_O	*Z* = 2
*M**_r_* = 294.30	*F*(000) = 312
Triclinic, *P*1	*D*_x_ = 1.371 Mg m^−^^3^
Hall symbol: -P 1	Mo *K*α radiation, λ = 0.7107 Å
*a* = 5.6859 (2) Å	Cell parameters from 400 reflections
*b* = 10.7190 (5) Å	θ = 1.0–28.0°
*c* = 12.1980 (5) Å	µ = 0.11 mm^−^^1^
α = 85.267 (3)°	*T* = 292 K
β = 83.990 (3)°	Plate, colorless
γ = 74.936 (4)°	0.38 × 0.24 × 0.15 mm
*V* = 712.76 (6) Å^3^	

### Data collection

**Table:**

Goniometer Xcalibur with Eos (Nova) detector diffractometer	2792 independent reflections
Radiation source: Enhance (Mo) X-ray Source	2109 reflections with *I* > 2σ(*I*)
graphite	*R*_int_ = 0.034
Detector resolution: 16.0839 pixels mm^-1^	θ_max_ = 26.0°, θ_min_ = 3.4°
ω scans	*h* = −7→7
Absorption correction: multi-scan (*CrysAlis PRO*; Oxford Diffraction, 2009)	*k* = −13→13
*T*_min_ = 0.951, *T*_max_ = 0.984	*l* = −15→15
18207 measured reflections	

### Refinement

**Table:**

Refinement on *F*^2^	Primary atom site location: structure-invariant direct methods
Least-squares matrix: full	Secondary atom site location: difference Fourier map
*R*[*F*^2^ > 2σ(*F*^2^)] = 0.037	Hydrogen site location: inferred from neighbouring sites
*wR*(*F*^2^) = 0.102	H atoms treated by a mixture of independent and constrained refinement
*S* = 1.09	*w* = 1/[σ^2^(*F*_o_^2^) + (0.046*P*)^2^ + 0.1184*P*] where *P* = (*F*_o_^2^ + 2*F*_c_^2^)/3
2792 reflections	(Δ/σ)_max_ < 0.000
201 parameters	Δρ_max_ = 0.19 e Å^−^^3^
0 restraints	Δρ_min_ = −0.14 e Å^−^^3^

### Special details

**Table:**

Experimental. CrysAlisPro, Oxford Diffraction Ltd., Version 1.171.33.34d Empirical absorption correction using spherical harmonics, implemented in SCALE3 ABSPACK scaling algorithm.
Geometry. All e.s.d.'s (except the e.s.d. in the dihedral angle between two l.s. planes) are estimated using the full covariance matrix. The cell e.s.d.'s are taken into account individually in the estimation of e.s.d.'s in distances, angles and torsion angles; correlations between e.s.d.'s in cell parameters are only used when they are defined by crystal symmetry. An approximate (isotropic) treatment of cell e.s.d.'s is used for estimating e.s.d.'s involving l.s. planes.
Refinement. Refinement of *F*^2^ against ALL reflections. The weighted *R*-factor *wR* and goodness of fit *S* are based on *F*^2^, conventional *R*-factors *R* are based on *F*, with *F* set to zero for negative *F*^2^. The threshold expression of *F*^2^ > σ(*F*^2^) is used only for calculating *R*-factors(gt) *etc*. and is not relevant to the choice of reflections for refinement. *R*-factors based on *F*^2^ are statistically about twice as large as those based on *F*, and *R*- factors based on ALL data will be even larger.

### Fractional atomic coordinates and isotropic or equivalent isotropic displacement parameters (Å^2^)

**Table:**

	*x*	*y*	*z*	*U*_iso_*/*U*_eq_	
H1W	0.159 (5)	0.640 (3)	0.786 (2)	0.096 (9)*	
H2W	0.059 (4)	0.773 (3)	0.799 (2)	0.096 (8)*	
O1	0.9249 (2)	−0.09595 (10)	0.89786 (9)	0.0468 (3)	
O4	0.4417 (2)	0.21103 (11)	0.32278 (8)	0.0479 (3)	
H4	0.5634	0.2371	0.3022	0.072*	
O3	−0.0136 (2)	0.32510 (10)	0.79355 (9)	0.0446 (3)	
C9	0.3842 (3)	0.14292 (13)	0.66438 (12)	0.0322 (3)	
C4	0.3717 (3)	0.11045 (13)	0.78842 (12)	0.0346 (3)	
H4A	0.2225	0.0817	0.8101	0.041*	
N2	0.5830 (2)	0.00283 (11)	0.81312 (10)	0.0400 (3)	
H2	0.6038	−0.0650	0.7766	0.048*	
C5	0.1804 (3)	0.34545 (15)	0.83520 (12)	0.0391 (4)	
C1	0.7456 (3)	−0.00217 (14)	0.88495 (12)	0.0352 (3)	
N1	0.7088 (2)	0.10279 (12)	0.94695 (10)	0.0416 (3)	
H1	0.8040	0.0987	0.9982	0.050*	
C3	0.3665 (3)	0.22455 (14)	0.85610 (12)	0.0348 (3)	
C12	0.4247 (3)	0.19198 (14)	0.43558 (12)	0.0366 (4)	
C13	0.5795 (3)	0.22773 (15)	0.50023 (12)	0.0396 (4)	
H13	0.6971	0.2686	0.4677	0.047*	
C14	0.5585 (3)	0.20247 (14)	0.61345 (12)	0.0378 (4)	
H14	0.6642	0.2261	0.6563	0.045*	
C11	0.2467 (3)	0.13405 (15)	0.48509 (13)	0.0407 (4)	
H11	0.1395	0.1117	0.4423	0.049*	
C2	0.5263 (3)	0.21559 (14)	0.93180 (12)	0.0368 (4)	
C10	0.2282 (3)	0.10937 (14)	0.59840 (13)	0.0379 (4)	
H10	0.1090	0.0696	0.6309	0.046*	
C6	−0.1877 (3)	0.43911 (17)	0.74982 (15)	0.0515 (4)	
H6A	−0.1987	0.5121	0.7938	0.062*	
H6B	−0.3484	0.4227	0.7537	0.062*	
O2	0.1978 (2)	0.45309 (11)	0.85073 (11)	0.0594 (4)	
C8	0.5280 (4)	0.31579 (16)	1.01034 (14)	0.0541 (5)	
H8A	0.3836	0.3856	1.0053	0.081*	
H8B	0.6701	0.3484	0.9917	0.081*	
H8C	0.5316	0.2776	1.0843	0.081*	
C7	−0.1064 (4)	0.47022 (19)	0.63274 (16)	0.0673 (6)	
H7A	−0.0706	0.3936	0.5920	0.101*	
H7B	0.0377	0.5015	0.6304	0.101*	
H7C	−0.2341	0.5355	0.6005	0.101*	
O5W	0.1583 (3)	0.71105 (16)	0.75335 (11)	0.0583 (4)	

### Atomic displacement parameters (Å^2^)

**Table:**

	*U*^11^	*U*^22^	*U*^33^	*U*^12^	*U*^13^	*U*^23^
O1	0.0563 (7)	0.0347 (6)	0.0454 (6)	0.0015 (5)	−0.0149 (5)	−0.0075 (5)
O4	0.0647 (8)	0.0479 (7)	0.0323 (6)	−0.0128 (6)	−0.0113 (5)	−0.0060 (5)
O3	0.0406 (6)	0.0380 (6)	0.0526 (7)	−0.0044 (5)	−0.0058 (5)	−0.0040 (5)
C9	0.0347 (8)	0.0270 (7)	0.0340 (8)	−0.0034 (6)	−0.0057 (6)	−0.0065 (6)
C4	0.0376 (8)	0.0309 (8)	0.0355 (8)	−0.0085 (6)	−0.0030 (6)	−0.0040 (6)
N2	0.0543 (8)	0.0273 (7)	0.0376 (7)	−0.0035 (6)	−0.0129 (6)	−0.0076 (5)
C5	0.0446 (9)	0.0366 (9)	0.0337 (8)	−0.0068 (7)	0.0009 (7)	−0.0055 (6)
C1	0.0470 (9)	0.0292 (8)	0.0282 (7)	−0.0077 (7)	−0.0026 (7)	−0.0009 (6)
N1	0.0540 (9)	0.0349 (7)	0.0351 (7)	−0.0043 (6)	−0.0139 (6)	−0.0075 (5)
C3	0.0422 (9)	0.0321 (8)	0.0291 (7)	−0.0082 (7)	0.0005 (6)	−0.0046 (6)
C12	0.0457 (9)	0.0292 (8)	0.0323 (8)	−0.0008 (7)	−0.0097 (7)	−0.0071 (6)
C13	0.0460 (9)	0.0399 (9)	0.0357 (8)	−0.0154 (7)	−0.0034 (7)	−0.0053 (6)
C14	0.0416 (9)	0.0406 (9)	0.0352 (8)	−0.0131 (7)	−0.0096 (7)	−0.0081 (6)
C11	0.0390 (9)	0.0409 (9)	0.0440 (9)	−0.0057 (7)	−0.0170 (7)	−0.0106 (7)
C2	0.0491 (9)	0.0305 (8)	0.0291 (7)	−0.0073 (7)	−0.0014 (7)	−0.0039 (6)
C10	0.0342 (8)	0.0362 (8)	0.0442 (9)	−0.0080 (7)	−0.0066 (7)	−0.0057 (7)
C6	0.0433 (10)	0.0427 (10)	0.0637 (11)	0.0012 (8)	−0.0094 (8)	−0.0089 (8)
O2	0.0660 (8)	0.0335 (7)	0.0786 (9)	−0.0046 (6)	−0.0223 (7)	−0.0095 (6)
C8	0.0767 (13)	0.0410 (10)	0.0427 (9)	−0.0033 (9)	−0.0164 (9)	−0.0146 (7)
C7	0.0882 (16)	0.0475 (11)	0.0607 (12)	−0.0030 (10)	−0.0195 (11)	−0.0001 (9)
O5W	0.0737 (10)	0.0535 (9)	0.0480 (8)	−0.0163 (7)	−0.0009 (7)	−0.0085 (7)

### Geometric parameters (Å, °)

**Table:**

O1—C1	1.2450 (18)	C12—C13	1.383 (2)
O4—C12	1.3712 (18)	C13—C14	1.384 (2)
O4—H4	0.8200	C13—H13	0.9300
O3—C5	1.3354 (19)	C14—H14	0.9300
O3—C6	1.4601 (19)	C11—C10	1.384 (2)
C9—C14	1.382 (2)	C11—H11	0.9300
C9—C10	1.388 (2)	C2—C8	1.500 (2)
C9—C4	1.523 (2)	C10—H10	0.9300
C4—N2	1.4710 (18)	C6—C7	1.493 (3)
C4—C3	1.523 (2)	C6—H6A	0.9700
C4—H4A	0.9800	C6—H6B	0.9700
N2—C1	1.3268 (19)	C8—H8A	0.9600
N2—H2	0.8600	C8—H8B	0.9600
C5—O2	1.2150 (19)	C8—H8C	0.9600
C5—C3	1.467 (2)	C7—H7A	0.9600
C1—N1	1.3662 (19)	C7—H7B	0.9600
N1—C2	1.3872 (19)	C7—H7C	0.9600
N1—H1	0.8600	O5W—H1W	0.83 (3)
C3—C2	1.343 (2)	O5W—H2W	0.93 (3)
C12—C11	1.382 (2)		
			
C12—O4—H4	109.5	C9—C14—C13	121.54 (14)
C5—O3—C6	116.65 (13)	C9—C14—H14	119.2
C14—C9—C10	117.91 (13)	C13—C14—H14	119.2
C14—C9—C4	120.72 (13)	C12—C11—C10	119.95 (14)
C10—C9—C4	121.32 (13)	C12—C11—H11	120.0
N2—C4—C9	108.75 (11)	C10—C11—H11	120.0
N2—C4—C3	109.61 (12)	C3—C2—N1	120.40 (13)
C9—C4—C3	113.46 (12)	C3—C2—C8	126.94 (14)
N2—C4—H4A	108.3	N1—C2—C8	112.60 (13)
C9—C4—H4A	108.3	C11—C10—C9	121.23 (14)
C3—C4—H4A	108.3	C11—C10—H10	119.4
C1—N2—C4	127.84 (12)	C9—C10—H10	119.4
C1—N2—H2	116.1	O3—C6—C7	109.76 (14)
C4—N2—H2	116.1	O3—C6—H6A	109.7
O2—C5—O3	122.53 (15)	C7—C6—H6A	109.7
O2—C5—C3	125.31 (15)	O3—C6—H6B	109.7
O3—C5—C3	112.14 (13)	C7—C6—H6B	109.7
O1—C1—N2	123.60 (13)	H6A—C6—H6B	108.2
O1—C1—N1	119.71 (14)	C2—C8—H8A	109.5
N2—C1—N1	116.69 (13)	C2—C8—H8B	109.5
C1—N1—C2	123.59 (13)	H8A—C8—H8B	109.5
C1—N1—H1	118.2	C2—C8—H8C	109.5
C2—N1—H1	118.2	H8A—C8—H8C	109.5
C2—C3—C5	120.99 (13)	H8B—C8—H8C	109.5
C2—C3—C4	121.53 (13)	C6—C7—H7A	109.5
C5—C3—C4	117.47 (13)	C6—C7—H7B	109.5
O4—C12—C11	118.08 (13)	H7A—C7—H7B	109.5
O4—C12—C13	122.33 (15)	C6—C7—H7C	109.5
C11—C12—C13	119.58 (14)	H7A—C7—H7C	109.5
C12—C13—C14	119.76 (15)	H7B—C7—H7C	109.5
C12—C13—H13	120.1	H1W—O5W—H2W	105 (2)
C14—C13—H13	120.1		
			
C14—C9—C4—N2	−71.27 (17)	C9—C4—C3—C5	52.91 (18)
C10—C9—C4—N2	106.19 (15)	O4—C12—C13—C14	−178.02 (14)
C14—C9—C4—C3	50.98 (18)	C11—C12—C13—C14	1.5 (2)
C10—C9—C4—C3	−131.56 (14)	C10—C9—C14—C13	−0.3 (2)
C9—C4—N2—C1	126.81 (16)	C4—C9—C14—C13	177.20 (13)
C3—C4—N2—C1	2.3 (2)	C12—C13—C14—C9	−0.6 (2)
C6—O3—C5—O2	10.0 (2)	O4—C12—C11—C10	177.99 (13)
C6—O3—C5—C3	−168.50 (13)	C13—C12—C11—C10	−1.6 (2)
C4—N2—C1—O1	−177.32 (14)	C5—C3—C2—N1	−176.83 (13)
C4—N2—C1—N1	2.8 (2)	C4—C3—C2—N1	2.9 (2)
O1—C1—N1—C2	174.44 (14)	C5—C3—C2—C8	5.9 (2)
N2—C1—N1—C2	−5.6 (2)	C4—C3—C2—C8	−174.35 (15)
O2—C5—C3—C2	26.2 (2)	C1—N1—C2—C3	2.9 (2)
O3—C5—C3—C2	−155.34 (14)	C1—N1—C2—C8	−179.51 (15)
O2—C5—C3—C4	−153.49 (16)	C12—C11—C10—C9	0.7 (2)
O3—C5—C3—C4	24.92 (18)	C14—C9—C10—C11	0.3 (2)
N2—C4—C3—C2	−5.05 (19)	C4—C9—C10—C11	−177.23 (13)
C9—C4—C3—C2	−126.82 (15)	C5—O3—C6—C7	85.59 (18)
N2—C4—C3—C5	174.68 (12)		

### Hydrogen-bond geometry (Å, °)

**Table:**

*D*—H···*A*	*D*—H	H···*A*	*D*···*A*	*D*—H···*A*
N1—H1···O1^i^	0.86	2.09	2.9411 (17)	171
O5W—H1W···O2	0.83 (3)	2.06 (3)	2.881 (2)	172 (3)
N2—H2···O4^ii^	0.86	2.14	2.978 (2)	165
O5W—H2W···O1^iii^	0.93 (3)	1.88 (3)	2.799 (2)	167 (2)
O4—H4···O5W^iv^	0.82	1.86	2.674 (2)	176

Symmetry codes: (i) −*x*+2, −*y*, −*z*+2; (ii) −*x*+1, −*y*, −*z*+1; (iii) *x*−1, *y*+1, *z*; (iv) −*x*+1, −*y*+1, −*z*+1.
